# Supplementary material for: Error-mitigated quantum gates exceeding physical fidelities in a trapped-ion system
Source: Nat Commun. 2020 Jan 30;11:587. doi: 10.1038/s41467-020-14376-z (PMC6992797; doi:10.1038/s41467-020-14376-z)
Supplement: Supplementary file 1 — Supplementary Information [file 41467_2020_14376_MOESM1_ESM.pdf]

**Supplementary Information for:**

# Error-Mitigated Quantum Gates Exceeding Physical Fidelities in a Trapped-Ion System

**Shuaining et al.**

[1] E. Knill, D. Leibfried, R. Reichle, J. Britton, R. B. Blakestad, J. D. Jost, C. Langer, R. Ozeri, S. Seidelin, and D. J. Wineland. Randomized benchmarking of quantum gates. *Phys. Rev. A*, 77:012307, Jan 2008.

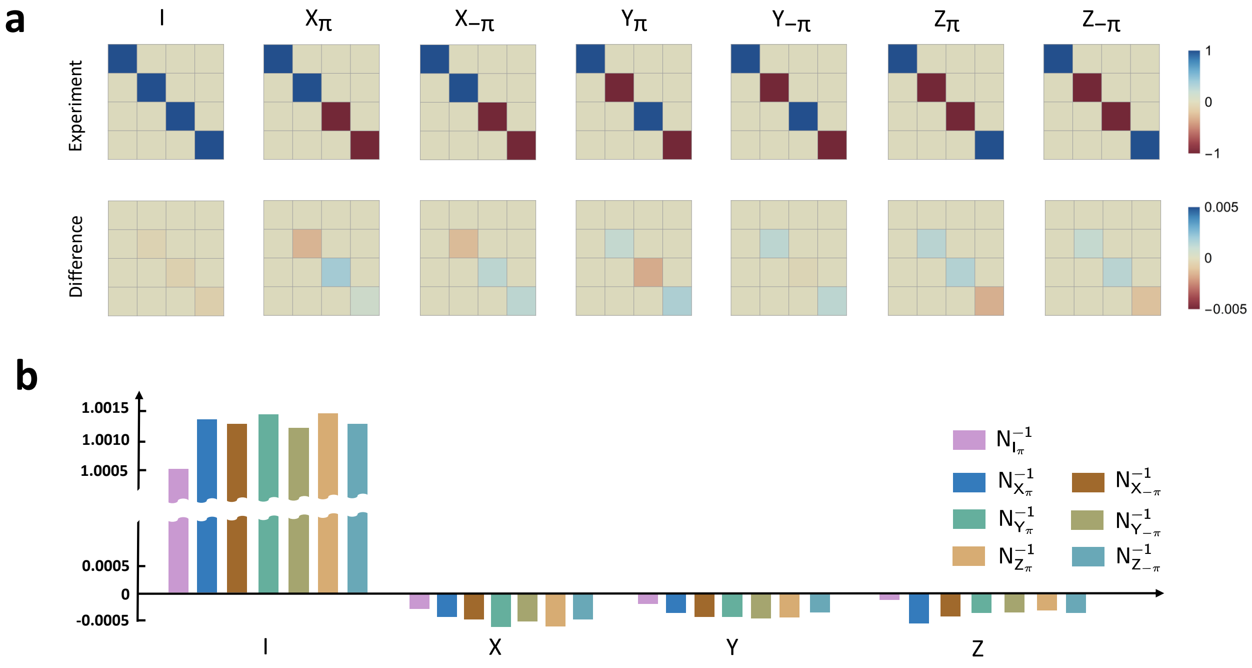


Supplementary Figure 1. **Characterization and decomposition of the experimental identity and $\pi$ pulses. a.** Single-qubit randomized benchmarking needs not only the computational gate set, but also the identity and $\pi$ pulses [1]. In order to implement error-mitigation for single-qubit randomized benchmarking, these gates should be characterized and the errors should also be decomposed. The upper row shows the experimentally-obtained PTMs and the lower row shows the difference between the experimental and the ideal matrices. **b.** Quasi-probabilities in the decomposition of the inverse noise operations of these single-qubit gates.

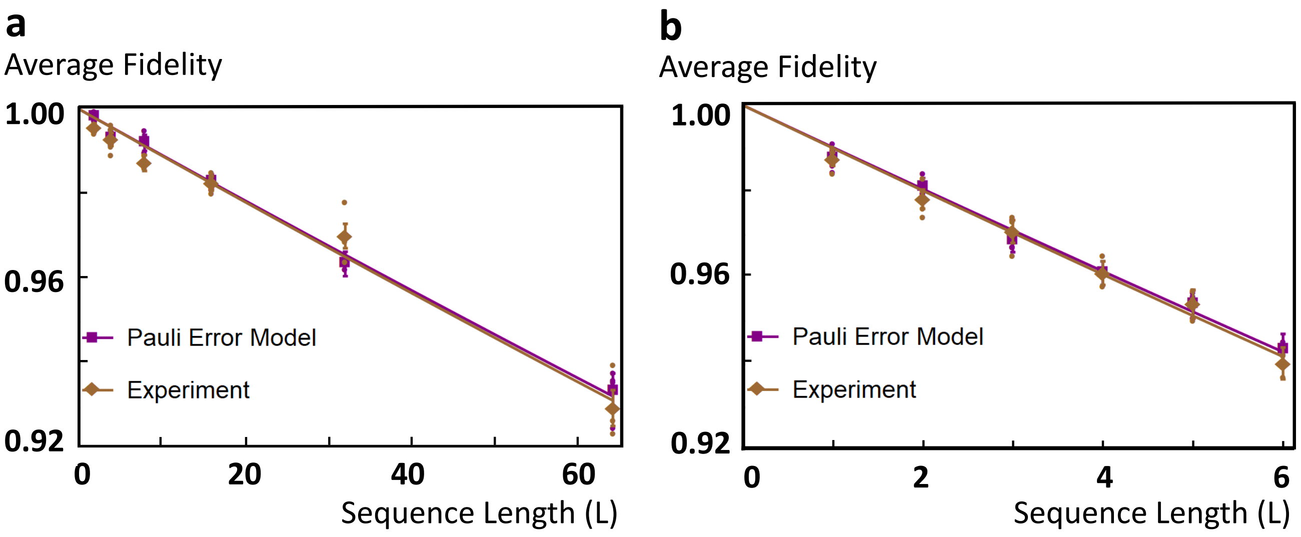


Supplementary Figure 2. **Verification of the Pauli-error assumption.** **a**. The average fidelity of the numerical (purple dots) and experimental (yellow dots) single-qubit random sequences as functions of the sequece length $L$. The numerical data are obtained by simulating the quantum dynamics with the experimental PTMs with the Pauli-error assumption. The error bars are the standard deviation of the average fidelities computed using the formula of uncertainty propagation. The curves are obtained by fitting an exponential decaying model to the data. The numerical and experimental error rates, being $(1.09 \pm 0.06) \times 10^{-3}$ and $(1.10 \pm 0.12) \times 10^{-3}$ respectively, are consistent within fitting errors. **b**. The same as **a**. for random two-qubit sequences. The numerical and experimental error rates are $(0.97 \pm 0.05) \times 10^{-2}$ and $(0.99 \pm 0.06) \times 10^{-2}$. Thus the comparison in **a**. and **b**. validate the Pauli-error assumption in our system.

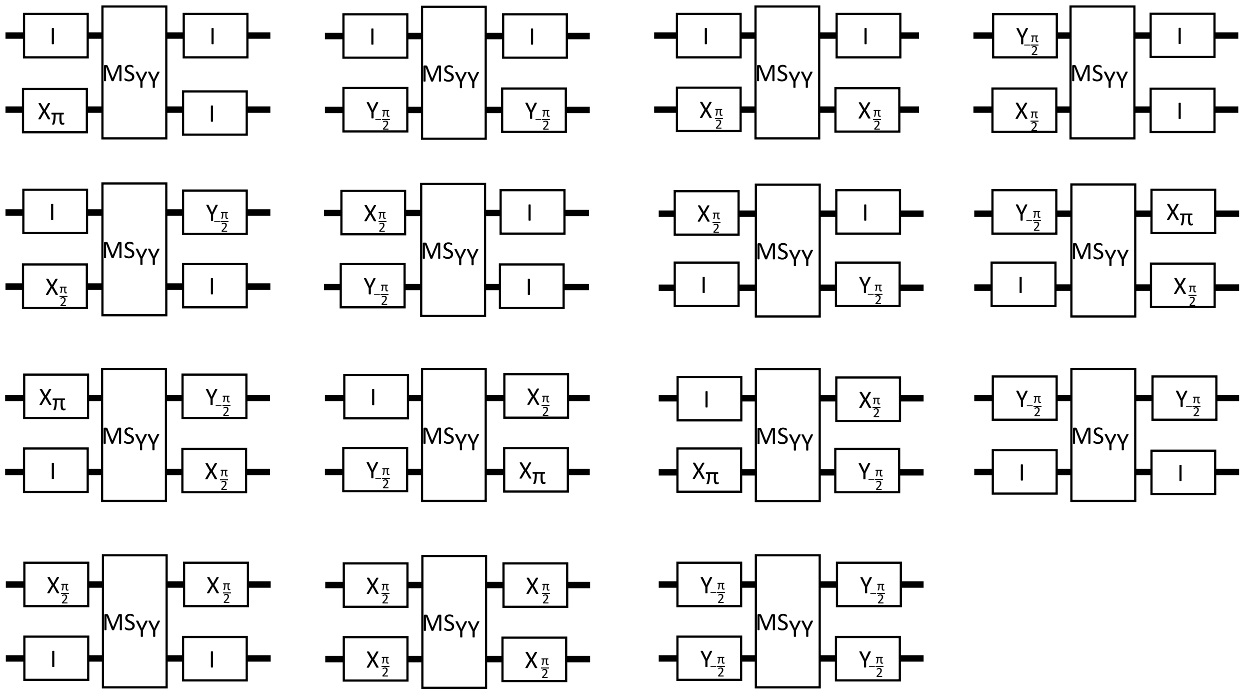


Supplementary Figure 3. **Experimental circuits for the characterization of the** $\mathrm{MS}_{YY}$ **gate.** The two-qubit system is first prepared in the initial $|00\rangle$ state by optical pumping. After implementing one of the quantum circuit, a projective measurement of $Z^{\otimes 2}$ is carried out. The above sequence is repeated 3000 times for each circuit to estimate the probability of the dark $|00\rangle$ state, which, together with the corresponding ansatz prediction, determines one of the Pauli-error rate for the experimental $\mathrm{MS}_{YY}$ gate.

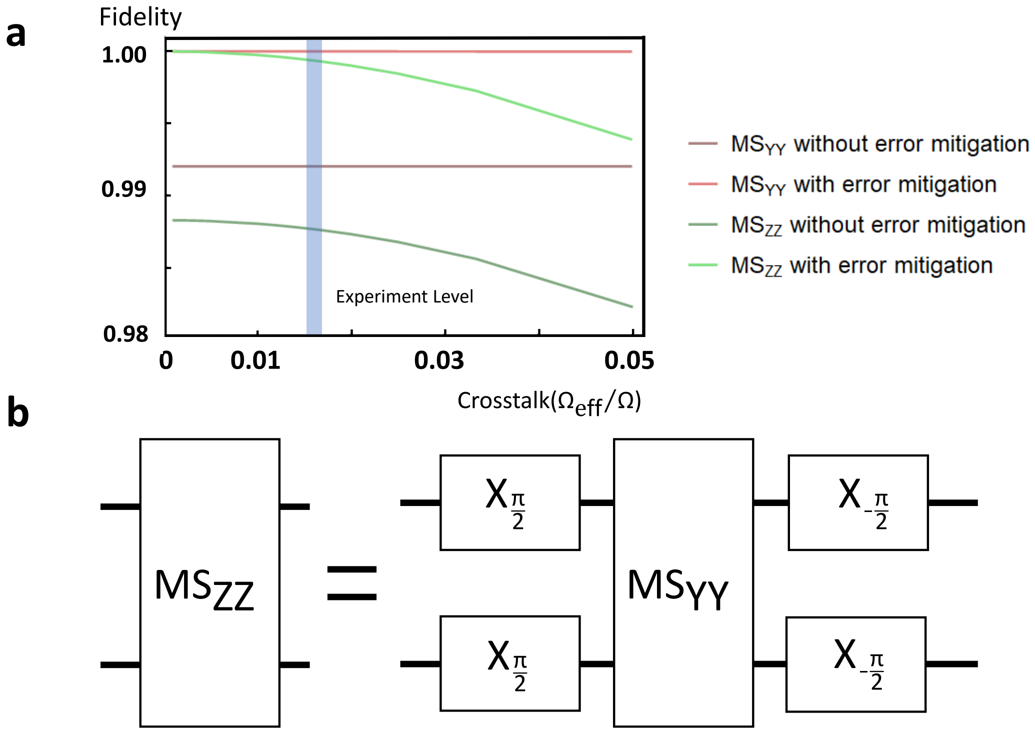


Supplementary Figure 4. **Analysis of the qubit crosstalk effect and the realization of** $\mathrm{MS}_{ZZ}$ **gate. a.** We numerically simulate the final-state fidelity of the original and error-mitigated $\mathrm{MS}_{YY}$ and $\mathrm{MS}_{ZZ}$ gates as functions of the qubit crosstalk strength, which is modeled by the ratio $\Omega_{\mathrm{eff}}/\Omega$, with $\Omega$ and $\Omega_{\mathrm{eff}}$ being the Rabi frequencies experienced by the target and the neighboring ions when a single-qubit gate is being implemented. The experimental level of the qubit crosstalk strength is shaded with blue, which given an estimation of $0.68 \times 10^{-3}$ for the residual error rate induced by the qubit crosstalk effect. **b.** The $\mathrm{MS}_{ZZ}$ gate is realized by adding single-qubit rotations before and after the $\mathrm{MS}_{YY}$ gate.
